# Investments in sexually transmitted infection research, 1997–2013: a systematic analysis of funding awarded to UK institutions

**DOI:** 10.7189/jogh.05.020405

**Published:** 2015-12

**Authors:** Michael G Head, Joseph R Fitchett, Jackie A Cassell, Rifat Atun

**Affiliations:** 1 University College London, Farr Institute for Health Informatics, London, UK; 2 London School of Hygiene & Tropical Medicine, Department of Clinical Research, Keppel Street, London, UK; 3 Harvard School of Public Health, Harvard University, Boston, USA; 4 Division of Primary Care and Public Health, Brighton and Sussex Medical School, Brighton, UK

## Abstract

**Background:**

We report the first study that analyses public and philanthropic investments awarded to UK institutions for research related to sexually transmitted infections (STIs).

**Methods:**

We systematically searched award data from the major funders for information on all infectious disease research funding awarded in 1997–2013. The STI–related projects were identified and categorised by pathogen, disease and type of science along the research pipeline from preclinical to translational research.

**Findings:**

We identified 7393 infection–related awards with total investment of GBP 3.5 billion. Of these, 1238 awards (16.7%) covering funding of GBP 719.1 million (20.5%) were for STI research. HIV as an STI received GBP 465 million across 719 studies; non–HIV STIs received GBP 139 million across 378 studies. The Medical Research Council provided greatest investment (GBP 193 million for HIV, GBP 45 million for non–HIV STIs). Preclinical awards totalled GBP 233 million (37.1%), whilst translational research received GBP 286 million (39.7%). Substantial proportions of HIV investment addressed global health research (GBP 265 million), vaccinology (GBP 110 million) and therapeutics (GBP 202 million). For other STIs, investments focused on diagnostics (GBP 45 million) and global health (GBP 27 million). Human Papilloma Virus research received GBP 58 million and chlamydia GBP 24 million. Funding for non–HIV STIs has declined in the three most recent years of this data set.

**Conclusions:**

The investment for HIV research awarded to UK institutions correlates with the high global burden, but other STIs are relatively neglected, including gonorrhoea and syphilis. Future STI funding should be better aligned with burden while addressing the emerging risk of antimicrobial resistance in *Neisseria gonorrhoeae* and outbreaks of other pathogens.

Sexually transmitted infections (STIs) are responsible for a large global burden of disease, of which HIV is individually the pathogen of greatest public health impact. In 2010, HIV accounted for 81.5 million disability–adjusted life years (DALYs), 3.3% of the global burden [1], whilst revised figures suggested an estimated 1.3 million deaths from HIV in 2013 [2].

There are approximately 500 million transmissions of STIs (other than HIV) worldwide annually, whilst seroprevalence of herpes simplex virus is highest in Africa, with infection found in 30–80% of women and 10% to 50% of men [3]. In the UK, high–risk human papilloma virus infection was detected in 15.9% of women [4]. Chlamydia is the most commonly diagnosed STI in England with over 200 000 new diagnoses in 2012 [5]. Stillbirth and neonatal damage due to congenital syphilis are thought to rival the early life burden of HIV infection, though arguably syphilis receives far less attention [6]. Incidence of syphilis is rising in many countries, including the UK and China [7,8]. There are over 100 million new cases of gonorrhoea globally each year [9], and incidence is also increasing in England [5]. The extent of observed antimicrobial resistance patterns has led to concerns that gonorrhoea will soon become untreatable [9,10]. Viral hepatitis and infection with *Mycoplasma genitalium* are further infections that add to the overall burden of STIs.

One tool in developing policies that attempt to better prevent, manage and treat all STIs is investment in research. Funding covers all types of science along the R&D research pipeline from pre–clinical to operational and implementation research. UK institutions have received an estimated GBP 2.6 billion of public and charitable funding to carry out infectious disease research between 1997 and 2010 [11], and estimates suggest the UK ranks second globally in terms of the amount of research and development (R&D) funding for neglected infectious disease research [12]. There are also 38 UK institutions in the most recent rankings list of the ‘top 100 most global universities’ [13]; thus there is a large quantity of research funding available for analysis coming from institutions carrying out relevant global activity. We report here on the funding for STI–related research awarded to UK institutions in 1997–2013, including three further years of investment data as part of an update on the previous work [11].

We identify probable areas of research strength and possible investment gaps in relation to global sexual health that will be of relevance to policy–makers, funders and researchers, and then briefly discuss how new approaches might help with managing burdens and allocating existing resources to the most appropriate preclinical, interventional or observation studies.

## METHODS

We analysed infectious disease–related studies funded over a 17–year period (1997–2013 inclusive) and awarded to UK institutions, and identified those relevant to STI research. Global health studies were defined as those which investigated diseases not endemic in the UK, or where the study had a clear reference to another country (eg, HIV in South Africa). We excluded open–access data from the pharmaceutical industry as it was limited and not representative.

The methods have been described in detail previously [11], and also to some extent replicated on the study website [14] and in other study publications [15-17]. The overarching data set was constructed by approaching the major sources of public and charitable funding for infectious disease research studies, including the Wellcome Trust, Medical Research Council and other research councils, UK government departments, the European Commission, Bill and Melinda Gates Foundation, and other research charities. Funders were identified by searching databases such as the National Research Register (now archived, ref. [18]), or Clinicaltrials.gov, authors knowledge of the funding landscape, through the knowledge of the Infectious Disease Research Network (www.idrn.org) and through searches of the internet. Where available, the funding decisions listed on their website were searched for infectious disease research awards (eg, Wellcome Trust); otherwise, the funder was directly approached and asked to provide information on their infection–related awards.

Each study was screened for relevance to infectious disease research and assigned to as many disease categories as appropriate. These included area of microbiology (bacteriology, virology, parasitology, mycology) and cross–cutting themes such as global health and antimicrobial resistance, as well as awards relating to new tools and products such as diagnostics, therapeutics and vaccines. The categories were selected based on author discussions during and since the data set was developed. Studies were also allocated to one of four categories (initially for 1997–2010 data) along the R&D pipeline: pre–clinical; phase 1, 2, or 3; intervention and product development; and translational research. For 2011–2013 data only, a fifth category has been added, this being cross–disciplinary, and is defined as a study significantly covering two types of science along the R&D pipeline (as per our categorisation above, also see ref. [14]. This category was added in response to a seemingly increasing number of awards involving consortia or programme grants that transcend the research pipeline boundaries of this study. The 1997—2010 data has not yet retrospectively been assessed for cross–disciplinary studies (capacity for a significant retrospective analysis of the entire initial data set is limited). The major funders were considered separately, while others were grouped into categories, such as professional bodies and societies, or other research charities. A total of 26 funder categories were used. All categorisation was carried out by author MGH, with provisional data sets circulated to authors for review and comment. Author JRF further verified a random sample of 10% of the 1997—2010 data set, whilst JRF and further external colleagues carried out similar process for 2011–2013 data. Author agreement was measured by a Kappa score (0.95 and 0.91) and differences settled by consensus. We excluded studies not immediately relevant to infection, veterinary infectious disease research studies (unless there was a clear zoonotic component), and studies which included UK collaborators, but where the funding was awarded to a non–UK institution. Unfunded studies were also excluded. Grants awarded in a currency other than pounds sterling were converted to UK pounds using the mean exchange rate in the year of the award. All awards were adjusted for inflation. Relative levels of investment were presented via a ‘GBP per disability–adjusted life years’ (DALY) figure; this represented the total investment in research per 1 DALY. The DALY figures were extracted from the 2010 Global Burden of Disease study [1].

This analysis includes HIV as an STI. Since sexual transmission of HIV is in most settings overwhelmingly the most common route, we included HIV–related studies as STI–related unless they specifically addressed other transmission modes. Therefore, studies investigating HIV via vertical transmission or via bloodborne pathways were excluded. Similarly, where other pathogens have multiple modes of transmission, eg, hepatitis B, they were only included if transmission by sexual contact were explicitly stated in the study title or abstract. Data management was carried out in Microsoft Excel and Access (versions 2007 and 2013) and statistical analysis with Stata (version 13).

## RESULTS

A total of 7393 awards were identified as relevant to all infectious diseases across 1997–2013 with a total investment of GBP 3.5 billion. Of these, 1238 awards (16.7%) were identified as relevant to STI research, with total funding of GBP 719.1 million (20.5% of all infectious disease funding; **Table 1**). Some top–level data reproduced here have been previously published as 1997–2010 results in an overview of all infectious disease funding (specifically study numbers and total funding for HIV including non–STI transmissions, gonorrhea, syphilis, chlamydia, HPV and HSV) [11]. There was one pre–clinical study in 2003 focusing on *Trichomonas vaginalis*.

**Table 1:** Total funding, mean and median award size of HIV and other sexually transmitted infections (STI) research awarded 1997–2013

Disease	Number of studies	Percentage of STI study number (%)*	Total funding (GBP)	Percentage of STI funding (%)*	Mean award, GBP (SD)	Median award, GBP (IQR)	Top funder, millions (%)
All STI studies	1238	n/a	719 086 641	n/a	580 845 (1 925 725)	144 138 (33 247–365 209)	MRC, 284.4 (39.5)
Non–HIV STIs	402	32.47	155 630 214	21.64	387 139 (965 424)	105 115 (17 827–251 356)	MRC, 45.2 (29.0)
HIV	873	70.52	596 800 543	82.99	66 3534 (2 213 359)	173 109 (39 374–454 801)	MRC, 192.8 (32.0)
Chlamydia	119	9.61	24 485 887	3.41	205 763 (556 606)	60 212 (11 450–180 498)	UK government department, non–DH, 9.6 (39.2)
Gonorrhoea	20	1.62	1 388 703	0.19	69 435 (96 071)	13 968 (3699–144 980)	Wellcome, 0.46 (33.3)
Syphilis	5	0.40	1 061 560	0.15	212 312 (152 848)	207 346 (113 088–229 907)	Wellcome, 0.57 (53.5)
Candida	87	7.03	29 458 307	4.10	338 601 (445 301)	261 386 (86 394–382 357)	BBSRC, 11.3 (38.5)
Mycoplasma	3	0.24	245 667	0.03	81 889 (107 412)	36 409 (46 989–204 559)	MRC, 0.20 (83.3)
HPV	164	13.25	58 254 838	8.10	355 212 (811 689)	113 852 (38 476–242 110)	Charity, 31.9 (54.8)
Herpes Simplex Virus	10	0.81	2 530 037	0.35	253 003 (381 987)	95 514 (15 682–309 610)	Wellcome, 2.0 (78.1)
Viral hepatitis	3	0.24	74 448	0.01	24 816 (24 446)	13 135 (8401–52 911)	Other, 0.05 (71.1)

n/a – not applicable, SD – standard deviation; IQR – interquartile range, MRC –Medical Research Council

*Percentages in are calculated as a fraction of all STI research. Because awards can cover more than one disease area or product category, the sum of these column percentages do not add up to exactly 100%.

**Figure 1:**
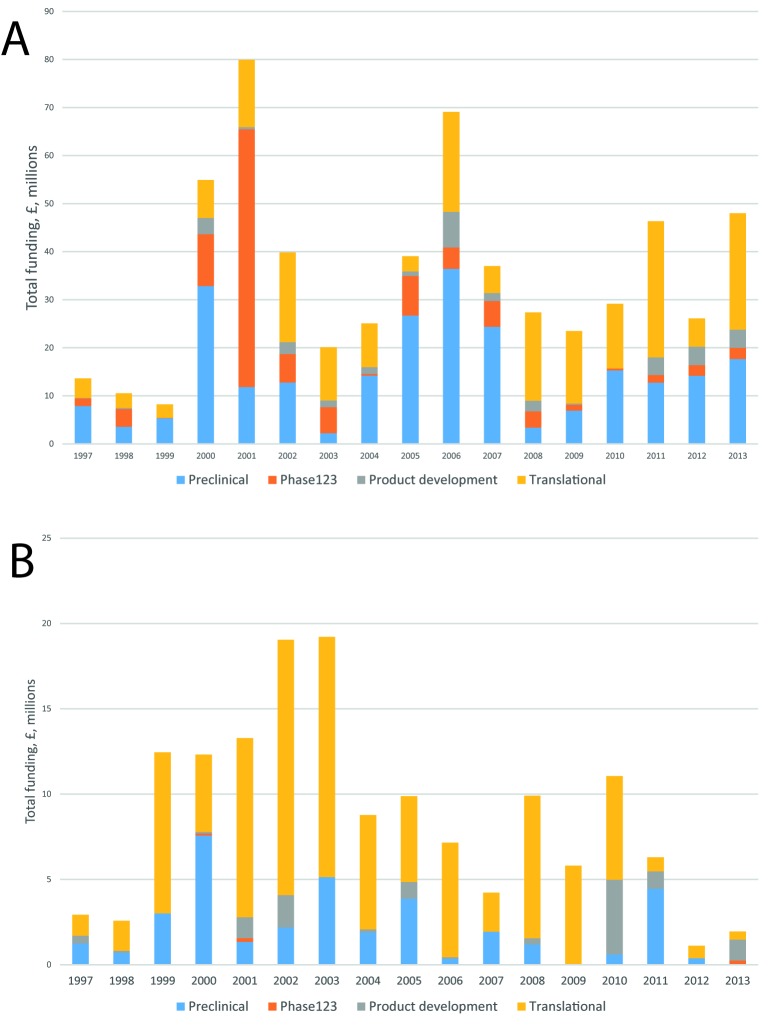
Investments on HIV (**A**) and other sexually–transmitted infection (**B**) research awarded to the UK over time and by type of science (x1 HIV and x2 STI cross–disciplinary studies not shown here).

Of this, GBP 596.8 million (83.0%) was related to HIV across 873 studies (70.5%), and GBP 155.6 million (21.6%) was invested in other STIs over 378 studies (32.5%). Median study funding for HIV research was GBP 173 109 (IQR GBP 39 374–454 801); median study funding for other STIs was GBP 105 115 (IQR GBP 17 827–251 356). A wide variety of funders contributed greatly to the sum funding, but the Medical Research Council invested the greatest amount for both HIV (GBP 192.8 million, 32.0%) and for other STIs (GBP 45.2 million, 29.0%). Annual funding is volatile with no consistent temporal trend in funding awards for either HIV or other STI research, and it appears as though funding for non–HIV STIs is declining in the most recent years of this data set (**Figure 1**).

For HIV research, pre–clinical science received GBP 247.8 million (41.5%) across 358 studies, phase I to III trials GBP 110.6 million (18.5%) across 62 studies, product development research GBP 32.9 million (5.5%) across 55 studies, and implementation and operational research GBP 194.4 million (32.6%) across 397 studies; there were also one cross–disciplinary study awarded between 2011–2013 (**Table 2**). For other STIs, pre–clinical science received GBP 35.9 million (23.1%) across 123 studies, phase I to III trials GBP 0.6 million (0.4%) across 4 studies, product development research GBP 12.0 million (7.7%) across 27 studies, and implementation and operational research GBP 99.5 million (64.0%) across 246 studies; Further, there were also three cross–disciplinary studies (two for non–HIV STIs, and one for HIV research) totalling GBP 18.7 million.

**Table 2:** Funding of research into HIV and other sexually transmitted infections (STIs) 1997–2013, described by type of science

Study type	All STI	non–HIV STI	HIV
**Pre–clinical:**			
Study numbers	461	123	358
Funding (GBP)	267 053 431	35 881 994	247 843 494
**Phase I – III:**			
Study numbers	62	4	62
Funding (GBP)	106 983 764	589 207	110 562 571
**Intervention & product development:**			
Study numbers	79	27	55
Funding (GBP)	40 749 294	11 967 782	32 949 526
**Translational:**			
Study numbers	633	246	397
Funding (GBP)	285 593 246	99 542 253	194 387 022

Within HIV research (**Table 3**), global health–related studies received GBP 264.9 million (44.4% of HIV research) across 228 studies (across all infectious disease, studies with a clear global health component represented 36.3% of all funding). There was also GBP 109.7 million (18.4%) invested in vaccinology, GBP 202.3 million (33.9%) in therapeutics and GBP 25.2 million (4.2%) in diagnostics. For other STI research, GBP 27.2 million was concentrated on global health (17.5% of all non–HIV STI funding), across 38 studies. The main focus here was for studies relating to non–pathogen–specific STI research; despite sum funding of GBP 24.5 million, there was just one study (GBP 0.3 million) considering chlamydia in a global context

**Table 3:** Funding of research into HIV and other sexually transmitted infections (STIs) described by general disease theme

Disease	Number of studies	Total funding (GBP)	Percentage of all HIV funding	Mean award, GBP (SD)	Median award, GBP (IQR)	Top funder, millions (%)
**HIV/AIDS:**						
Global health	228	264 900 733	44.4%	1 161 845 (3 885 828)	198 934 (53 409–694 934)	DFID, 78.9 (29.8)
Vaccinology	70	109 708 029	18.4%	1 567 258 (2 963 178)	558 247 (256 053–1 361 466)	European Commission, 29.5 (26.9)
Therapeutics	184	202 317 448	33.9%	1 099 551 (4 089 456)	195 947 (36 809–672 265)	European Commission, 60.1 (29.7)
Paediatrics	77	32 503 928	5.4%	422 128 (635 597)	196 270 (47 595–464 190)	MRC, 7.6 (44.0)
Diagnostics	41	25 173 418	4.2%	613 985 (1 797 939)	82 787 (14 835–410 458)	MRC, 4.3 (17.3)
Antimicrobial resistance	33	20 773 195	3.5%	629 490 (1 775 416)	125 119 (59 327–236 201)	European Commission, 8.9 (42.7)
Primary care	19	4 452 155	0.7%	234 323 (401 638)	49 368 (11 333–282 707)	Wellcome, 2.7 (61.5)
Economics	7	1 143 190	0.2%	163 312 (134 121)	82 872 (70 853–234 309)	Wellcome, 0.4 (30.7)
Behavioural science	20	3 849 174	0.6%	192 458 (122 607)	195 533 (109 439–308 732)	MRC, 2.8 (72.2)
**Non–HIV sexually–transmitted infections:**						
Global health	38	27 186 347	17.5%	715 430 (1 184 356)	289 717 (70 843–737 257)	DFID, 12.5 (46.1)
Vaccinology	14	3 537 045	2.3%	252 646 (325 470)	116 207 (62 976–237 470)	Department of Health, 1.2 (34.6)
Therapeutics	11	3 793 661	2.4%	344 878 (331 566)	242 343 (144 138–320 031)	Charity, 2.1 (54.5)
Paediatrics	12	1 217 304	0.8%	101 442 (204 546)	319 72 (14 098–80 664)	MRC, 0.7 (60.6)
Diagnostics	123	44 705 952	28.7%	363 463 (931 522)	72 293 (11 793–175 234)	Charity, 15.5 (34.5)
Antimicrobial resistance	5	5 741 870	3.7%	1 148 374 (2 472 306)	6470 (776–165 259)	DFID, 5.6 (97.0)
Primary care	41	4 988 181	3.2%	121 663 (248 283)	18 389 (11 450–172 042)	Department of Health, 2.4 (48.7)
Economics	6	2 573 574	1.7%	428 929 (550 030)	201 856 (131 999–514 066)	Department of Health, 2.3 (91.9)
Behavioural science	18	3 260 456	2.1%	181 136 (128 084)	181 211 (98 963–247 826)	MRC, 1.8 (56.3)

SD – standard deviation; IQR – interquartile range, MRC – Medical Research Council, DFID – Department for International Development

There was GBP 3.5 million (2.3%) invested in in vaccinology research, GBP 3.8 million (2.4%) for therapeutics and GBP 44.7 million (28.7%) for diagnostics. Antimicrobial resistance–related investments were GBP 20.7 million (3.5%) for HIV, and GBP 5.7 million (3.7%) for other STIs.

Where data are available and presented, the global burden of disease, measured in disability adjusted years (DALYs), was correlated with levels of research investment (**Table 4**). Time periods were chosen to reflect the years in which burden data was available. From 2004 DALYs, there is an overall investment of GBP 10.20 per DALY for HIV research, and GBP 14.93 per DALY for other STIs. Furthermore, there was investment of GBP 6.53 per DALY for chlamydia research, and relatively less investment in syphilis (GBP 0.37) and gonorrhoea (GBP 0.39). Using 2010 burden data, which is the most recent time period for which complete burden data was available, the relative investments against burden are – HIV GBP 7.33, other STIs GBP 14.18, chlamydia GBP 34.29, syphilis GBP 0.11 and gonorrhoea GBP 4.92. Annual investment over time increases for HIV research, but noticeably decreases for research of other STIs (**Figure 1**).

**Table 4:** Comparisons between investment in HIV and other sexually transmitted infections (STIs) research and global burden of disease

Disease	Number of studies	Total funding (GBP)	DALY 2004	DALY 2010	Total investment relative to 2004 burden (GBP per DALY)	Total investment relative to 2010 burden (GBP per DALY)	Annual Investment 1997–2004 (GBP)	Annual Investment 2005–2010 (GBP)	Annual investment 2011–2013 (GBP)
**HIV**	873	596 800 543	5 851 2843	81 457 000	10.20	7.33	30 178 341	37 300 762	43 856 416
**non–HIV STIs:**	402	155 630 214	10 424 871	10 978 000	14.93	14.18	11 322 986	8 005 387	5 671 334
Chlamydia	119	24 485 887	3 748 198	714 000	6.53	34.29	2 182 799	706 665	927 836
Gonorrhoea	20	1 388 703	3 549 975	282 000	0.39	4.92	96 047	30 004	146 768
Syphilis	5	1 061 560	2 846 113	9 578 000	0.37	0.11	96 930	47 686	0
Candida	87	29 458 307	n/a	n/a	n/a	n/a	1 259 942	2 117 066	2 225 459
Mycoplasma	3	245 667	n/a	n/a	n/a	n/a	5139	34 093	0
HPV	164	58 254 838	n/a	n/a	n/a	n/a	4 489 759	2 723 738	1 998 113
Herpes simplex virus	10	2 530 037	n/a	n/a	n/a	n/a	2 171 198	782 286	1 054 630
Viral hepatitis	3	74 448	n/a	n/a	n/a	n/a	9306	0	0

DALY – disability–adjusted life year, HPV – human papilloma virus, n/a – not applicable

## DISCUSSION

Our study is the first systematic analysis of research funding for STI research, including STI–related HIV, awarded to UK institutions. Over the 17–year time period of the study, there is consistent funding for HIV research along the entire research pipeline in all types of science, including phase I–III trials. However, this is not replicated for other STIs where much research is categorised as translational research and there are fewer preclinical studies. HIV studies are typically larger in size, and HIV received almost four times as much funding as other STIs combined. Within HIV, global health and therapeutics studies received most investment, whilst other STI studies focused on global health and diagnostics. Non–HIV STIs broadly experienced a decline in annual research investments over time, and relative to global burden, syphilis and gonorrhoea are relatively less well funded than HIV and chlamydia. Total funding per annum is unpredictable.

A global HIV research infrastructure is now well established, and this is partly so because of the formation of UNAIDS, an over–arching well–funded independent body that has successfully encouraged investment, collaborative work and sustained political leadership [19,20]. There are other groups tracking specific aspects of global HIV research funding [21], so the research gaps may be less obvious than in other disease areas. Substantial public and philanthropic investments have been directed towards the development of an HIV vaccine, shown both within this UK analysis here and also in international projects [21]. This global quest has proven relatively fruitless so far but has potentially very high impact should the goals be achieved. Preventive measures may be the most effective approach in the long–term, and there are widespread efforts to research and develop microbicides [21] and understand how best to implement effective behaviour change [22,23]. Having closely observed the UK portfolio of HIV research, it is arguably the large scale behavioural science studies that are most lacking by comparison with the USA and Global South, as well as how to maximise the effectiveness of genitourinary medicine clinics and other services in primary care that offer HIV testing. Research may also focus on gaining a better understanding of how to increase testing in high risk groups such as UK men who have sex with men, a key population which continues to experience HIV incidence comparable with generalised epidemics.

Antimicrobial resistance (AMR) is a global threat, and has historically been under–funded in the UK [24]. There have been few new antibacterial therapeutics in recent years and there are several reasons for this, including the pharmaceutical industry perceiving a lack of return on their investment compared to long–term chronic illnesses [25] resulting in market failure [26]. The levels of resistance in *Neisseria gonorrhoeae* are exceptionally high [9], and the organism has long been known for its exceptional ability to evolve resistance genes. There is virtually no UK research focussing on gonococcal AMR, although clinical trials in the US are investigating the potential of Solithromycin, a 4th generation macrolide with promising results in a phase II trial [27]. This is a critical area of potential research focus for funders and policymakers to consider, particularly in light of the 2014 review of the global economics of AMR [28].

The investments into chlamydia research are relatively strong when compared with global burdens, but this infection is the most common STI in the UK [5]. The vast majority was translational in focus and very little was categorized as global health. It may be that a much larger proportion of the research was considered to address local needs, as opposed to other infections like HIV with the significant emphasis on global health. Much HPV–related research was either pre–clinical in nature or had a focus on diagnostic and screening programmes. This approach may well change now that effective vaccines have been implemented into the UK immunisation schedules – modelling the effectiveness of vaccine programmes and research into increasing uptake and future cervical screening programmes may now take priority. Syphilis research may best centre on areas such as development of a vaccine [29], how best to implement behaviour change at the preventive level and how to ensure access to treatments for those who need it. We identified only one study *for T.* vaginalis, despite its being described as the most common curable STI in the world with implications for increased HIV transmission [30].

It is important that researchers have access to a diverse group of funding institutions, to ensure broad based investments for different areas of STI research, and to increase predictability. There is evidence that where public sector investment decreases, so does private investment [31]. Thus incentives for, and collaborations with, the private sector are important for the research environment as a whole. New sources of investment would help with the focus on priority areas. Should greater investment be secured, it will need to be spent wisely on research that clearly adds to the evidence base, does not unnecessarily duplicate existing work or knowledge, and will be high impact (measuring impact will vary depending upon the type of science addressed in the research). A coordinated proactive approach between existing funders, and international co–operations where required, would help further identify and fund priority areas, and international systematic analyses similar to that reported here could be replicated to provide detailed information on the current and historical funding landscape in other countries. Future linkage between investment and outputs of research such as publications, impact on policy and products such as databases would give some indication of the power and quality of research.

Our study has several limitations, which have been highlighted and discussed in detail elsewhere [11]. There was little publicly–available data from the pharmaceutical industry. Hence, there is a data gap particularly in relation to funding of clinical trials and the development of vaccines and diagnostics, which the pharmaceutical and biotechnology industry are mostly financing (the sums of public and charitable investment in HIV–related phase I–III trials are not replicated across most other disease areas including other STIs). Beyond disease burden, other measures, such as economic burden should also be utilised when prioritising limited resources, but little information is available regarding the economic impact of STIs. We rely on the original data being complete and accurate, and are unable to take into account distribution of funds from the lead institution to collaborating partners or any annualisation of the total funding awarded, nor can we assess quantity of each award given to overheads or the impact of the introduction of full–economic costing. Also, assigning studies to categories is a subjective and imperfect process – although we used at least two researchers to do this to reduce inter–observer error. Our study focuses on UK–led investments – we do not know if similar patterns (eg, a lack of public or charitably–funded clinical trials in STIs) would also emerge if the analysis were repeated for other high–income countries, and we do not know how globally representative the UK investments are against other countries portfolios. We have not here measured either the outputs or impact of funded research. The assessment against measures of burden used the most comprehensive DALY figures available, but they are only estimates and their reliability is not precisely known; there may also be definitional differences between data sets and burden data was not available for all infections.

This analysis of UK investments in STI research highlights some areas of probable research strength, particularly with global health–related studies and more generally across the HIV research pipeline. It also suggests there are clear gaps and a need for greater research into syphilis, gonorrhoea and antimicrobial resistance. Work is ongoing to produce in–depth analyses of infectious disease research investments awarded to US institutions, and this will allow comparisons with UK strengths and weaknesses and help to set benchmarks for assessing investment vs disease burden. There is a continuing need to extend beyond this to build a global funding database of all types of HIV and other STI–related research. This analysis can be of use for funders, policymakers and researchers and act as a stimulus for targeting priority areas in STI research.
